# Diagnostic reasoning in rehabilitation nutrition: Position paper by the Japanese Association of Rehabilitation Nutrition (secondary publication)

**DOI:** 10.1002/jgf2.549

**Published:** 2022-04-28

**Authors:** Hidetaka Wakabayashi, Keisuke Maeda, Ryo Momosaki, Yoji Kokura, Yoshihiro Yoshimura, Dai Fujiwara, Shintaro Kosaka, Norio Suzuki

**Affiliations:** ^1^ Department of Rehabilitation Medicine Tokyo Women's Medical University Hospital Tokyo Japan; ^2^ Department of Geriatric Medicine National Center for Geriatrics and Gerontology Obu Japan; ^3^ Department of Rehabilitation Medicine Mie University Graduate School of Medicine Mie Japan; ^4^ Department of Nutritional Management Keiju Hatogaoka Integrated Facility for Medical and Long‐term Care Nanao Japan; ^5^ Center for Sarcopenia and Malnutrition Research Kumamoto Rehabilitation Hospital Kumamoto Japan; ^6^ Department of Rehabilitation Medicine Saka General Hospital Shiogama Japan; ^7^ Department of Internal Medicine Nerima Hikarigaoka Hospital Tokyo Japan; ^8^ Division of Cardiology, Department of Internal Medicine St. Marianna University School of Medicine Kawasaki Japan

**Keywords:** analytic reasoning, anorexia, nonanalytic reasoning, sarcopenia, weight loss

## Abstract

Diagnostic reasoning is the thought process used to arrive at a diagnosis based on symptoms, examination findings, and laboratory values. Diagnosis is categorized as nonanalytic reasoning (intuition) and analytic reasoning (analysis). Rehabilitation nutrition involves the diagnosis of nutritional disorders, sarcopenia, and excess or deficient nutrient intake. There is usually only one correct answer for the presence or absence of these. On the other hand, there may be no single correct answer for the causes of anorexia, weight loss, or sarcopenia, and analytical reasoning is required. In this case, diagnostic reasoning involves hypotheses. Simply using nutritional supplements without performing diagnostic reasoning about these causes is like prescribing antipyretic analgesics to a patient with a headache without diagnosing the cause of the headache. To maximize function and quality of life in rehabilitation nutrition, it is necessary to suspect the common causes of anorexia, weight loss, and sarcopenia in all cases.

## 1 INTRODUCTION: WHAT IS DIAGNOSTIC REASONING?

The rehabilitation nutrition care process includes a rehabilitation nutrition assessment and diagnostic reasoning.^1^ The rehabilitation nutrition assessment and diagnostic reasoning involves a holistic assessment using the International Classification of Functioning, Disability and Health, medical history, evaluation of nutritional status and activity level, and assessment of sarcopenia/frailty to inform diagnostic reasoning about nutritional status, sarcopenia, excess or deficiency in nutrient intake, and their causes. Insufficient diagnostic reasoning naturally leads to inadequate rehabilitation nutrition care processes after the diagnosis, making it difficult to provide interventions that maximize function and quality of life. Therefore, it is necessary to master diagnostic reasoning to practice rehabilitation nutrition effectively.

Diagnostic reasoning is the thought process used to arrive at a diagnosis based on symptoms, examination findings, and laboratory values. The thought process is classified into nonanalytic reasoning (intuition, system 1) and analytic reasoning (analytic, system 2). Nonanalytic reasoning is a diagnostic method in which the characteristic patterns of disease are understood and recognized subconsciously and intuitively in a short period of time. On the other hand, analytic reasoning is a diagnostic method in which a list of etiologies is created in the process of hypothesis formation, and prioritization is determined by frequency and severity. For example, a patient with a BMI of 14 would be intuitively diagnosed with undernutrition using primarily nonanalytic reasoning. In contrast, a patient with a BMI of 20 would not be intuitively diagnosed with undernutrition but would go through a series of hypotheses and their testing to arrive at a diagnosis. Both nonanalytic and analytic reasoning should be used in a well‐balanced manner in both cases.

The causes of anorexia, weight loss, and sarcopenia are particularly important for diagnostic reasoning in rehabilitation nutrition. Although these causes can sometimes be adequately diagnosed by nonanalytic reasoning alone, they often require analytic reasoning. Adopting a small, high‐energy, high‐protein diet or using oral nutritional supplements without applying diagnostic reasoning about the causes is like prescribing only antipyretic analgesics to a patient suffering from chronic headache without diagnosing the cause of the headache. It may work, but it does not solve the underlying problem.

Diagnosing whether a patient has undernutrition, sarcopenia, or frailty is not difficult because the criteria can be simply applied. However, analytic reasoning to examine multiple possible differential diagnoses and make a reasoned choice is required. In addition, multiple iatrogenic and noniatrogenic causes may be recognized. Therefore, mastering rehabilitation nutrition diagnostic reasoning requires daily case review and experience. In this position paper, we clarified our position, as the Japanese Association of Rehabilitation Nutrition, on the existing knowledge on undernutrition, overnutrition, causes of weight loss, sarcopenia, nutrient under‐intake, causes of anorexia, and nutrient over‐intake, together with diagnostic reasoning processes in cases, individual differences in diagnostic reasoning, and the process of learning diagnostic reasoning.

## 2 DIAGNOSTIC REASONING FOR UNDERNUTRITION

By nonanalytic reasoning, we can diagnose undernutrition when we see a person who is extremely thin. However, there are some individuals who are not diagnosed with undernutrition by the Global Leadership Initiative on Malnutrition (GLIM) criteria^2^ because they are thin, but healthy and have no etiologies such as decreased food intake, malabsorption, or inflammation. In addition, it is difficult to intuitively diagnose undernutrition in obese patients with a BMI of 25 or more who have complications of undernutrition.

In analytic reasoning, the following steps using the GLIM criteria are applied.

Step (1) A validated nutrition screening tool determines that the patient is at risk for undernutrition.

Step (2) A diagnosis of undernutrition is made when one or more of the following applies to the phenotypic criteria and etiologic criteria, respectively.

### 2.1 Phenotypic criteria

- Unintentional weight loss: This applies to weight loss of 5% or more within the past 6 months and 10% or more beyond 6 months.
- Low body mass index (BMI): BMI is less than 18.5 for Asian patients under 70 years of age, and BMI is less than 20 for those over 70 years of age. For non‐Asians, BMI is less than 20 for those under 70 years of age and BMI less than 22 for those over 70 years old.
- Low muscle mass: The Asian Working Group for Sarcopenia (AWGS) 2019 criteria^3^ are used for Asians. In the case of calf circumference, low muscle mass is defined as less than 34 cm in men and less than 33 cm in women.

### 2.2 Etiologic criteria

- Reduced food intake or assimilation: A decrease in food intake of 50% or less for more than 1 week, any decrease in food intake for more than 2 weeks, or any chronic gastrointestinal malabsorption.
- Inflammation related to acute disease, trauma, or chronic disease.

Step (3) The severity of low nutrition (whether it is moderate or severe) is determined.

The causes of undernutrition are classified as follows: chronic disease with inflammation, acute disease or trauma with severe inflammation, chronic disease with little or no inflammation, and starvation without inflammation (due to a food shortage caused by socioeconomic or environmental factors). When multiple causes are identified, the most influential cause is considered. In the case of starvation, the clinician should move on to search for the cause of nutrient under‐intake.

## 3 DIAGNOSTIC REASONING FOR OVERNUTRITION

Overnutrition in the rehabilitation nutrition diagnosis is a condition in which the patient is at risk of developing health problems or declining activities of daily living (ADLs) due to excessive fat accumulation. By nonanalytic reasoning, we can diagnose overnutrition if we see a person who is extremely obese. However, overnutrition is not diagnosed in athletes with high skeletal muscle mass and low body fat mass, even if they have a BMI of 25 or higher. In addition, it is difficult to diagnose overnutrition by intuition if the patient has a standard body type with a BMI in the normal range.

Using analytic reasoning, the clinician should evaluate body fat mass to identify excessive fat accumulation. If the visceral fat area is greater than 100 cm^2^ on an abdominal computed tomography (CT) image at the umbilical region, a diagnosis of overnutrition can be made. Bioelectrical impedance analysis (BIA) or dual‐energy x‐ray absorptiometry (DXA) can be employed to diagnose overnutrition when an individual's body fat percentage is greater than 25% in men and 32% in women, depending on age. Physical measurements may also be used: overnutrition can be diagnosed when the abdominal circumference is 85 cm or more for men and 90 cm or more for women.

Causes of overnutrition are categorized as excessive energy intake, insufficient energy expenditure, decreased basal metabolic rate due to aging or other causes, and diseases (endocrine disorders such as Cushing syndrome and hypothyroidism, and hereditary diseases such as Prader‐Willi syndrome). When multiple causes are present, the most influential cause should be considered.

## 4 DIAGNOSTIC REASONING FOR CAUSES OF WEIGHT LOSS

Weight loss is often diagnosed when an individual loses more than 5% of their body mass in 6–12 months, or more than 2 kg in 6 months. Nonanalytic reasoning is more likely to diagnose decreased food intake due to anorexia as the cause. In patients with advanced cancer, it is easy to intuitively diagnose cancer cachexia. However, weight loss may occur without anorexia or decreased food intake. Multiple causes may be present.

Analytical reasoning confirms the OPQRST mnemonic^4^ (Onset, Palliative & Provoke, Quality & Quantity, Region, Symptoms, Time course) of weight loss (Table 1). For a comprehensive analysis of the causes of weight loss, the Meals on Wheels mnemonic (Table 2)^5^ is useful. Causes of weight loss in rehabilitation nutrition are shown in Table 3. Common causes should be suspected in all cases. Multiple causes and drugs may be recognized for weight loss.

**TABLE 1:** The OPQRST mnemonic of weight loss

Onset: “When did weight loss begin?”
Palliative & Provoke: “Has weight loss continued/improved?”
Quality & Quantity: “How much weight loss?”
Region: “Where did you lose weight?”
Symptoms: “What other symptoms do you have? Is there anorexia, nausea/vomiting, dysphagia, constipation, diarrhea, taste disorder, olfactory disorder, general fatigue, dyspnea, pain, fever, depression, anxiety, muscle weakness, or decreased ADLs?”
Time course: “How is the progress after weight loss?”

Abbreviation: ADL, activities of daily living.

**TABLE 2:** The meals on wheels mnemonic

M: Medication
E: Emotional
A: Alcoholism, Abuse, Anorexia
L: Late‐life paranoia
S: Swallowing problems
O: Oral problems
N: Nosocomial infections, no money
W: Wandering
H: Hypothyroidism, hyperglycemia
E: Enteral problems
E: Eating problems
L: Low salt, low cholesterol
S: Stones, shopping problems, social problems, isolation

**TABLE 3:** Causes of weight loss in rehabilitation nutrition

**Common causes**
Malignancy
Gastrointestinal disease
Depression
Pharmaceutical (SGLT2 inhibitors, laxatives, NSAIDs, anti‐dementia drugs, antipsychotics, antidepressants, anticholinergics, anticancer drugs, diuretics, etc.)
Dysphagia (including disorders of oral function and environment)
Inappropriate nutritional management in hospitals and institutions
Dietary preferences in hospital/facilities (including swallowing adjusted diets)
**Frequent causes**
Chronic heart failure
Chronic respiratory failure (e.g., chronic obstructive pulmonary disease)
Chronic renal failure
Diabetes (including excessive dietary restrictions)
Electrolyte abnormalities (e.g., hypercalcemia and hyponatremia)
Dementia, delirium, disorders of life rhythm
Taste and smell disorders
Eating, cooking, and shopping require assistance
Social problems (e.g., economic deprivation and isolation)
Aging
**Rare causes**
Chronic infectious diseases (e.g., infective endocarditis and tuberculosis)
Collagen and autoimmune diseases
Neuromuscular diseases (Parkinson disease, amyotrophic lateral sclerosis, etc.)
Adrenocortical insufficiency
Hyperthyroidism

Abbreviations: NSAID, nonsteroidal anti‐inflammatory drug; SGLT2, sodium‐glucose cotransporter‐2.

## 5 DIAGNOSTIC REASONING FOR SARCOPENIA

By nonanalytic reasoning, sarcopenia can be diagnosed if the patient is thin, requires assistance with ADLs, and has low skeletal muscle mass and low physical function. However, the cause of low skeletal muscle mass and low physical function may be an undiagnosed neuromuscular disorder rather than sarcopenia.

Analytic reasoning uses the AWGS 2019 criteria.^3^ The diagnostic process in primary health care or community settings includes case finding, evaluation, and intervention, in that order. Case finding includes three items: the calf circumference measurement, the strength, assistance walking, rise from a chair, climbing stairs, and falls (SARC‐F) questionnaire, and the SARC‐F combined with calf circumference (SARC‐CalF). For the evaluation of sarcopenia, the individual's handgrip strength is measured as a muscle strength assessment, and a five‐chair stand test is performed as a physical function assessment. If the handgrip strength is less than 28 kg in men and less than 18 kg in women, the patient is considered to have low muscle strength, and if the five‐chair stand test is longer than 12 s, the patient is considered to have a low physical function. Low muscle strength or low physical function is diagnosed as possible sarcopenia.

For hospital and clinical research purposes, muscle strength is assessed by handgrip strength. Physical function is assessed either by the five‐chair stand test, 6‐meter gait speed (<1 m/s), or Short Physical Performance Battery (SPPB, <9 points). The diagnostic criteria for low muscle mass are the same as in the AWGS 2014.

The causes of sarcopenia are categorized as aging, activity, nutrition, disease, and iatrogenic. All causes are common in rehabilitation nutrition, and while multiple causes are often present, the cause with the greatest impact is considered. In the case of activity, clinicians should assess the duration and extent of low activity. In the case of nutrition, they should move to the diagnostic reasoning of nutrient under‐intake. In the case of disease, clinicians should assess the presence and extent of acute or chronic inflammation. In the case of an iatrogenic cause, they should assess the possibility of low activity due to inappropriate bed rest or food abstinence, undernutrition due to inappropriate nutritional care management, and illness or adverse drug events^1^.

## 6 DIAGNOSTIC REASONING FOR NUTRIENT UNDER‐INTAKE

In rehabilitation nutrition, a deficit of carbohydrates and fats is not diagnosed as nutrient under‐intake when the energy intake has been intentionally reduced by aggressive nutrition therapy aimed at weight loss. However, protein and micronutrient under‐intake should be noted. Nonanalytic reasoning would diagnose nutrient under‐intake in inpatients and facility residents who consistently leave more than half of their provided diet. On the other hand, nutrient under‐intake may be due to insufficient energy in the meal provided or too much physical activity, even if the patient consumes the entire meal provided.

In analytical reasoning, nutrient intake is calculated as the sum of diet plus enteral nutrition plus parenteral nutrition. Energy consumption is calculated using the Dietary Reference Intakes for Japanese, medical guidelines, and with the following formula: basic energy consumption multiplied by the activity coefficient multiplied by the stress coefficient. Next, intake minus consumption is used to diagnose the presence and degree of nutrient under‐intake. At that time, the presence or absence of an accumulated amount (deficient amount) due to aggressive nutritional therapy is checked.

The causes of nutrient under‐intake can be categorized as inadequate energy intake, impaired absorption due to disease or drugs, or increased energy consumption. Multiple causes may be present. In the case of energy under‐intake, clinicians should consider the presence or absence of anorexia and its cause if food intake is inadequate. If enteral nutrition or parenteral nutrition is deficient, they should consider whether it is intentional or unintentional. If absorption is impaired due to disease or drugs, clinicians should consider the exact causative disease and drug. In the case of increased energy expenditure, they should consider whether it is due to too much physical activity, involuntary movements, increased muscle tone, acute or chronic inflammation, etc.

## 7 DIAGNOSTIC REASONING FOR ANOREXIA

For the assessment of anorexia, the Simplified Nutritional Appetite Questionnaire (SNAQ)^6^, ^7^ can be used. Nonanalytic reasoning often leads clinicians to believe that an older person's inability to eat adequately is inevitable due to aging. Although aging is one cause of anorexia^8^, rehabilitation nutrition often recognizes causes of anorexia other than aging, some of which can be improved.

Analytic reasoning confirms the OPQRST mnemonic of anorexia (Table 4). The causes of anorexia in rehabilitation nutrition are listed in Table 5. Common causes should be suspected in all cases. Multiple causes and drugs may be recognized for anorexia.

**TABLE 4:** The OPQRST mnemonic of anorexia

Onset: “When did anorexia begin?”
Palliative & Provoke: “When does anorexia become stronger/weaker?”
Quality & Quantity: “How much anorexia? What can you eat?”
Region: “What do you think is wrong with your appetite?”
Symptoms: “What other symptoms do you have? Nausea/vomiting, pain, taste, smell?”
Time course: “What is the course of events after anorexia?”

**TABLE 5:** Causes of anorexia in rehabilitation nutrition

**Common causes**
Drug‐related (pregabalin, tramadol and other opioids, laxatives, NSAIDs, anti‐dementia drugs, antipsychotics, antidepressants, anticholinergics, steroids, antihistamines, anticancer drugs, bisphosphonates, Parkinson disease medications, muscle relaxants, diuretics, etc.)
Depression (including drug‐induced)
Dysphagia (including deterioration of oral function and environment, including drug‐related)
Dementia, delirium, disorders of life rhythm (including drug‐induced)
Gastrointestinal disorders (vomiting, diarrhea, constipation, functional dyspepsia, etc., including drug‐induced)
Hospital/facility food preferences (including adjusted swallowing diets)
**Often Causes**
Cachexia (malignancy, chronic heart failure, chronic respiratory failure, chronic renal failure, chronic infection, collagen disease, etc.)
Taste and smell disorders
Acute inflammation (e.g., acute infection)
Diabetes mellitus
Hypothyroidism
Aging (e.g., decreased activity, changes in eating, and exercise habits)

Abbreviation: NSAID, nonsteroidal anti‐inflammatory drug.

## 8 DIAGNOSTIC REASONING FOR NUTRIENT OVER‐INTAKE

In rehabilitation nutrition, nutrient over‐intake is not diagnosed when energy intake is intentionally increased by aggressive nutrition therapy aimed at weight gain. Based on nonanalytic reasoning, clinicians suspect nutrient over‐intake when inpatients and facility residents consume a large amount of food brought in, in addition to consuming a full provided meal. On the other hand, even if the patients do not consume the entire meal provided, they may have nutrient over‐intake due to excessive energy in the meal provided or too little physical activity. Analytic reasoning of nutrient over‐intake is similar to that for nutrient under‐intake.

The causes of nutrient over‐intake can be categorized as excessive energy intake, increased appetite due to disease or drugs, or inadequate energy expenditure. In the case of excess energy intake, the possibility of excess dietary intake should be examined. If enteral nutrition or parenteral nutrition is excessive, clinicians should consider whether it is intentional or unintentional. In the case of disease‐ or drug‐induced increased appetite, they should consider the causative disease or drug. If there is inadequate energy expenditure, clinicians should consider whether it is due to low physical activity, the effect of muscular hypotonia, hypothyroidism, etc.

## 9 REHABILITATION NUTRITION DIAGNOSTIC REASONING IN PRACTICE

As an example of applying diagnostic reasoning, we describe the case of an 83‐year‐old woman with a fracture of the proximal femur. She had a height of 153 cm, a normal body weight of 45 kg, and a normal BMI of 19.2. Her body weight at presentation was 41 kg, and her BMI was 17.5.

The woman's medical history was as follows. She lived alone and was independent in ADLs but had physical frailty. She had a history of hypertension and chronic constipation. She fell at home, suffered a right hip fracture, and was hospitalized. Right artificial head replacement surgery was performed the next day. She was transferred to a convalescent rehabilitation hospital on the 15th day after the injury with the intention of being discharged home. One week after admission to the convalescent rehabilitation hospital, the patient's food intake was about 40% whole gruel meal (1500 kcal, 60 g protein), and the rehabilitation nutrition team intervened because anorexia and weight loss (1 kg in the first week after admission) were observed.

The patient's blood pressure was 110/60 mmHg, pulse 74 bpm, body temperature 36.1°C, and respiration 14 times/min. She had no pain, including at the fracture site. Defecation frequency was once a day. She experienced nausea but no vomiting and no taste disturbance. She was dentally edentulous with full dentures. She experienced no delirium at night.

Her laboratory results were as follows: WBC 5.2 × 10^3^/mm^3^, RBC 304 × 10^4^/mm^3^, Hb 9.3 g/dl, Plt 16.3 × 10^4^/mm^3^, TP 5.5 mg/dl, Alb 2.8 g/dl, AST 17 IU/L, ALT 15 IU/L, ALP 103 IU/L, LDH 154 IU/L, γ‐GTP 26 IU/L, BUN 11 mg/dl, Cr 0.54 mg/dl, Na 131 mEq/L, K 4.3 mEq/L, Cl 97 mEq/L, Ca 9.0 mg/dl, CRP 0.62 mg/dl.

Her medications were: amlodipine (5) 1T1x, lubiprostone (24) 2T2x, tramadol (50) 2T2x, sulpiride (50) 3T3x, risperidone (1) 1T1x, eldecalcitol (0.75) 1T1x, omeprazole (10) 1T1x.

Her scores related to ADLs were: Barthel Index 55 points and Revised Hasegawa Dementia Scale (HDS‐R) 23 points. Her calf circumference was 26 cm, her handgrip strength was 14 kg, and her basal energy expenditure was 941 kcal (Harris‐Benedict formula).

The patient was doing 8–9 units (160–180 min) of physical therapy (PT) per day, but she was easily fatigued and was often in a sitting position; she was mostly bedridden except for PT and mealtimes.

Table 6 shows the results of the eight authors' responses to the following questions for this case. (1) What additional information would you like to collect to support your diagnostic reasoning regarding anorexia and weight loss? (2) What are the most likely causes of anorexia and weight loss (first, second, third)? (3) How should clinicians intervene for the first cause of anorexia and weight loss? (4) How should clinicians intervene for the second cause of anorexia and weight loss? (5) How should clinicians intervene for the third cause of anorexia and weight loss? (6) Please suggest nutrition goals after 1 month. (7) Please suggest rehabilitation goals after 1 month. (8) Please recommend nutrition intervention methods. (9) Please recommend rehabilitation intervention methods.

**TABLE 6:** Responses regarding case

Respondents	What additional information would you like to collect to support your diagnostic reasoning regarding anorexia and weight loss?	What are the most likely causes of anorexia and weight loss (first, second, third)?	How should clinicians intervene for the first cause of anorexia and weight loss?	How should clinicians intervene for the second cause of anorexia and weight loss?	How should clinicians intervene for the third cause of anorexia and weight loss?	Please suggest nutrition goals after 1 month.	Please suggest rehabilitation goals after 1 month.	Please recommend nutrition intervention methods.	Please recommend rehabilitation intervention methods.
A	Presence or absence of depression Presence and cause of feeding and swallowing difficulties (including denture maladjustment) Presence or absence of Parkinson's syndrome When was the drug used started? Preference for hospital food	No. 1: Drug‐induced nausea (lubiprostone, tramadol) No. 2: Drug‐induced Parkinson's syndrome and dysphagia (sulpiride, risperidone) No. 3: Depression	Discontinue lubiprostone and tramadol. Use magnesium oxide and acetaminophen if constipation and pain are present.	Discontinue sulpiride and risperidone. If insomnia is present, use sleeping pills (e.g., Suvorexant). If nocturnal delirium develops, use yokukansan or yokukansan‐ka‐tinpihange.	Use antidepressants (e.g., mirtazapine). Prescribe occupational therapy and add psychological occupational therapy.	1 kg weight gain if nausea, Parkinson's syndrome, and dysphagia improve after discontinuing lubiprostone, tramadol, sulpiride, and risperidone.	If nausea, parkinsonism, and dysphagia improve after discontinuing lubiprostone, tramadol, sulpiride, and risperidone, the patient will be able to walk short distances independently with T‐cane and have light assistance with bathing and stairs.	Discontinue lubiprostone, tramadol, sulpiride, and risperidone. Change to an adjusted swallowing diet that is appropriate for the patient's level of dysphagia and preferences. Add medium chain triglyceride oil and protein powder to whole gruel. Add oral nutritional supplements that are appropriate for the patient's level of dysphagia and preferences.	Discontinue lubiprostone, tramadol, sulpiride, and risperidone. Physical therapy should be light‐loaded to prevent disuse for the time being, and increased to include resistance training once nutritional intake improves. Prescribe occupational therapy and add psychological occupational therapy. Add swallowing therapy and provide swallowing function training. Denture adjustment by dentistry.
B	Findings on extrapyramidal symptoms Patient's subjective view of food patterns When did you lose weight or food intake, along with medication history? CT or MRI of the brain Identification of inactive delirium	No. 1: Drug‐induced anorexia No. 2: Inactive delirium No. 3: Vicious cycle of frailty	Consider a dose reduction or withdrawal of tramadol and risperidone (possible cause of disorientation/inactive delirium). Consider withdrawal of sulpiride (possible extrapyramidal symptoms). Consider withdrawal of omeprazole (possible malabsorption/constipation). Consider changing lubiprostone (possible gastrointestinal symptoms such as abdominal bloating). Food fortification and oral nutritional supplements (Sip feed, Medication Pass), dietary appearance considerations.	Provide care related to adjusting the living environment and diurnal rhythm adjustment. Consider reducing or withdrawing at‐risk medications. Food fortification and oral nutritional supplements (Sip feed, Medication Pass), dietary appearance considerations.	Food fortification and oral nutritional supplements (Sip feed, Medication Pass), and dietary appearance considerations. Plan increases in physical activity and exercise in conjunction with increased nutritional intake.	Dietary intake of more than 1800 kcal/day, weight recovery of 2 kg, handgrip strength of 18 kg.	One‐on‐one rehabilitation with a therapist for a caloric intake of at least 1800 kcal/day and at least 2 h/day. Barthel Index 80 points.	Food fortification and oral nutritional supplements (Sip feed, Medication Pass), provide dietary appearance considerations. Provide 1900 kcal/day and 75 g protein. Conduct case conferences with rehabilitation staff and nurses to share information on activity levels.	Increase or decrease activity levels to match nutritional intake. Individualized review of necessary activities in the home for rehabilitation intervention. Conduct a multidisciplinary case conference including a dietitian every 2 weeks. Share information on nutrition goals and rehabilitation goals.
C	When to start each drug Characteristics of stools Abdominal characteristics (visual and palpation) Swallowing while eating Oral hygiene status	No. 1: Drug‐induced anorexia No. 2: Drug‐induced dysphagia No. 3: Gastrointestinal tract diseases	Drug dose reduction (tramadol).	Drug reduction (sulpiride, risperidone).	Abdominal imaging examination.	Weight gain (about 2 kg).	Independent in walking and ADLs.	Consider snacking and oral nutritional supplements.	Adjust the amount of gait training according to nutritional status.
D	Type of food for side dishes Availability of low‐sodium diets Oral condition (e.g., do dentures fit well?) Blood glucose level When to prescribe tramadol	No. 1: Inappropriate food texture No. 2: Unnecessarily low‐sodium diets No. 3: Side effects of tramadol	Review whether the food texture is suitable.	Discontinue if on a low‐sodium diet.	Withdrawal or change of tramadol dose.	1 kg weight gain.	Barthel Index gait 10 points.	Check the condition of the dentures and assess whether the diet texture is suitable. Offer a non‐sodium diet. If food intake does not increase, consider parenteral nutrition.	ADL training is the basis of the program, and if the patient is able (or has the prospect of being able) to consume the target energy level, resistance training and endurance training should be used to increase muscle mass.
E	Presence of depression and/or delirium Oral condition (including denture fit) Presence or absence of gastrointestinal disease (including a close examination of constipation) Social and family environment Thyroid function	No. 1: Drug‐induced No. 2: Environmental changes No. 3: Dementia and/or depression	Propose reduction or discontinuation of the causative drug, change to another drug, or nonpharmacological treatment.	Environmental adjustments: adjusting life rhythm, adjusting eating location (e.g., dining room instead of hospital room), encouraging getting out of bed during the day, meeting with family and friends.	Search for causes of dementia and depression and treat if possible.	1 kg weight gain.	Indoor walking independence with walking aids.	Low‐dose, high‐energy diets, oral nutritional supplements, peripheral parenteral nutrition.	Gait training, muscle strengthening training of the whole body, and training for activities of daily living while ensuring that nutritional management is appropriate.
F	Drug prescription history (how the current drug being used was prescribed) Presence and degree of depression Sleeping conditions at night Denture fit Dietary intake and preferences prior to illness	No. 1: Drug‐induced (nausea with tramadol, hypersedation with risperidone, abdominal distention with lubiprostone, anorexia with eldecalcitol or omeprazole, etc.) No. 2: Decreased general endurance No. 3: Denture incompatibility	Tramadol and risperidone prescriptions should be discontinued. Consider the need for prescription adjustments for other medications.	Increase time away from bed outside of rehabilitation and meals.	Adjustment of dentures.	The patient should gain 2 kg (4.3 lb) in 1 month. The patient should be able to consume all of the provided regular diet.	Handgrip strength of 18 kg (AWGS sarcopenia threshold) or greater. Can walk with a cane in the ward with supervision. Independently defecate in the toilet.	Nutritional administration should be set up with an activity coefficient of 1.3, a stress coefficient of 1.0, and an energy accumulation of 500 kcal. The target energy intake should be 1720 kcal and 65 g protein (protein energy ratio 15%). Three meals should be provided and supplemented with additional oral nutritional supplements after rehabilitation and exercise.	Defecation is performed by guiding the patient to the toilet. Since improvement in nutritional status cannot be expected while the nutritional intake is low, resistance training should be avoided, and basic ADL training and walking training should be implemented. Once nutritional intake is sufficient, nutritional status is expected to improve, so the amount of gait training in rehabilitation should be increased, stair climbing training should be added, and resistance training (chair stand training) should be performed in the hospital ward.
G	Pre‐admission diet Dietary preferences Hobbies Last defecation HDS‐R score details	No. 1: Drug‐induced No. 2: Discrepancy in diet (texture and preference) No. 3: Iatrogenic malnutrition during hospitalization	Discontinuation of risperidone, tramadol, and lubiprsotone.	Providing meals that meet the preferences, oral environment, and assisting swallowing function.	Snacking with oral nutritional supplements, nutrient loading with a high‐energy diet such as power rice (added medium chain triglyceride oil and protein powder) or tube feeding.	Continuation of nutritional administration of at least 2000 kcal and 60 g of protein per day with a target body weight of 45 kg.	Extended standing retention time, target Barthel Index 70–80 points	The patient should be placed on a nasogastric tube, and enteral nutrition should be administered combined with oral intake. The patient should be transitioned to a high‐energy diet as she gains strength.	First, reduce supine time by using wheelchair rides and other activities during the day, and second, implement high‐load rehabilitation when the patient has increased endurance through extended rehabilitation time.
H	Dietary intake prior to hospitalization Start date of each drug Preferences for meals served Eating and swallowing function (including denture fit) Stool characteristics (volume, color)	No. 1: Malignancy No. 2: Drug‐induced No. 3: Dietary preference issues	Malignant tumor: After close examination by CT, endoscopy, fecal occult blood, tumor markers, etc., surgical and endoscopic treatment, chemotherapy and radiation therapy should be considered. If there is an obstruction of the gastrointestinal tract, we also aim to improve the obstruction.	Drug‐related: Discontinue the suspect drug or switch to a drug with the same effect that is less likely to cause anorexia and nausea.	Dietary preference issues: consider changing or adjusting the meal content and flavor to the individual's preferences. If the salt content is restricted for hypertension, consider relaxing the restriction.	Improve oral intake and prevent further weight loss. If oral intake is sufficient to meet energy requirements, the goal is to gain at least 1 kg.	Increase time for gait training with supervision; spend time in a sitting position outside of PT and meal times.	Assuming elimination of the cause and improvement. Energy requirement: basic energy consumption 941 kcal x activity coefficient 1.2 = 1129 kcal/day; current intake: calculated to be approximately 600 kcal/day. There is a high risk of refeeding syndrome (BMI < 18.5, weight loss of more than 10% within 3–6 months). Increase by 5–10 kcal/kg/day (approximately 200–400 kcal/day), and once the energy requirement is satisfied, increase by 1450–1700 kcal/day (daily energy accumulation 250 kcal to increase the dose up to 500 kcal/day). If improvement takes time or is difficult due to malignancy or other factors, add oral nutritional supplements concomitantly or in divided doses.	Until energy requirements are met, light‐load rehabilitation should be focused on lengthening sitting time, lengthening ADL time, etc. to prevent further muscle strength and ADL decline due to low activity. Once the energy requirement is met and energy accumulation can be added, gait training and low‐load resistance training should be increased.

The most frequent responses for additional information to be collected were oral, denture, and swallowing function (7 respondents), time of drug initiation (6 respondents), eating pattern (4 respondents), food preferences (4 respondents), depression (3 respondents), defecation/constipation (3 respondents), delirium (2 respondents), and extrapyramidal symptoms (2 respondents). The most likely cause of anorexia and weight loss was drug‐related according to 6 respondents, and 2 others included it within the top three causes. 2 respondents identified drug‐related causes in the top two causes. Other causes included inappropriate eating patterns, food preferences, and depression, each of which was ranked in the top three by 2 respondents. The intervention methods varied depending on the cause, but since most of the interventions were drug‐related, many of the interventions were related to reviewing medications. The nutritional goal after 1 month was a 1–2 kg body weight gain, and the rehabilitation goals after 1 month were to be able to walk indoors independently or with supervision and to have a Barthel Index of 70–80 points. Daily energy requirements ranged from 1450 to 1900 kcal. Rehabilitation intervention methods were mostly tailored to nutritional intake.

## 10 INDIVIDUAL DIFFERENCES IN REHABILITATION NUTRITION DIAGNOSTIC REASONING

The cause of anorexia and weight loss in this case is most likely to be drug‐related. In addition, oral, denture, and swallowing problems, inappropriate eating patterns, food preferences, and depression may be complicating factors. Since these are common causes of anorexia and weight loss in rehabilitation nutrition, their presence should be suspected, and information gathered in all cases. Other possibilities that should be considered and information collected include gastrointestinal disease, dementia, malignancy, the vicious cycle of frailty, hypoactive delirium, and environmental changes. Note that when a robust patient visits an outpatient department of internal medicine or general practice with a primary concern of anorexia or unintended weight loss, the common causes are malignancy, gastrointestinal disease, or depression. The frequency of common causes of anorexia and weight loss varies by setting.

With the exception of drug‐related causes, there were large individual differences in clinicians' diagnostic reasoning regarding the causes of anorexia and weight loss. It is not surprising that differences in occupation, setting, and years of experience may lead to some individual differences in diagnostic reasoning regarding the causes of anorexia and weight loss. However, if the diagnosis of drug‐induced anorexia and weight loss had not been made in this case, this would clearly be problematic. If the diagnosis of drug‐induced anorexia and weight loss were not made in this case, it would be difficult to maximize the patient's function and quality of life by adjusting eating patterns, providing oral nutritional supplements, and providing split meals and snacks. Therefore, in rehabilitation nutrition, when a patient presents with anorexia and weight loss, it is necessary to suspect the presence of common causes in all cases and to carry out analytical reasoning.

## 11 MASTERING REHABILITATION NUTRITION DIAGNOSTIC REASONING

The acquisition of rehabilitation nutrition diagnostic reasoning requires daily case review and experience. With limited clinical experience, it is easy for clinicians treating anorexia and weight loss to simply use oral nutritional supplements without performing diagnostic reasoning about the cause of these symptoms. When sarcopenia is observed, it is easy to use resistance training or oral nutritional supplements without performing diagnostic reasoning about the cause. To avoid such shortcuts, it is useful to have discussions through case studies at multidisciplinary rehabilitation nutrition conferences. However, the discussion of nutritional disorders, sarcopenia, and the presence or absence of excess or deficient nutrient intake is not sufficient. Since there is only one correct answer for these, the significance of the discussion among multidisciplinary staff members is minimal. The ability to make diagnostic reasoning can be gradually improved through case studies in which the causes of anorexia, weight loss, and sarcopenia are discussed by multidisciplinary staff members. It is important to pursue these causes further by discussion, and appropriate diagnostic reasoning will lead to higher quality rehabilitation nutrition goal setting and rehabilitation nutrition intervention.

## 12 CONCLUSION

Some rehabilitation nutrition questions have a single correct answer, while others may have multiple correct answers. There is usually a single correct answer for the diagnostic reasoning regarding the presence or absence of undernutrition, overnutrition, sarcopenia, and excess or deficiency in nutrient intake. However, when diagnostic criteria are updated, the updated diagnostic criteria should be used. On the other hand, there may not be a single correct answer for the causes of anorexia, weight loss, or sarcopenia. In this case, diagnostic reasoning includes creating hypotheses and rehabilitation nutrition goal setting.^9^ However, to maximize function and quality of life, the most likely hypothesis should be considered from the outset. Therefore, the clinician must suspect the common causes of anorexia and weight loss as hypotheses in all rehabilitation nutrition cases. In that case, not only nonanalytical reasoning but also analytical reasoning should be used while collecting information related to the causes. If clinicians' ability of nonanalytical reasoning can also be improved by repeating analytical reasoning with multidisciplinary colleagues through case reviews, they will achieve more appropriate rehabilitation nutrition goal setting and rehabilitation nutrition intervention skills. This is the secondary English version of the original Japanese manuscript for “Diagnostic reasoning in rehabilitation nutrition: position paper by the Japanese Association of Rehabilitation Nutrition.”^10^

## CONFLICT OF INTEREST

The authors have stated explicitly that there are no conflicts of interest in connection with this article.
